# Collagen Cross-Linking Lignin Improves the Bonding Performance of Etch-and-Rinse Adhesives to Dentin

**DOI:** 10.3390/ma15093218

**Published:** 2022-04-29

**Authors:** Diego Martins de Paula, Diego Lomonaco, Antônio Moisés Parente da Ponte, Karen Evellin Cordeiro, Madiana Magalhães Moreira, Massimo Giovarruscio, Salvatore Sauro, Victor Pinheiro Feitosa

**Affiliations:** 1 Paulo Picanço School of Dentistry, R. Joaquim Sá, 900-Dionísio Torres, Fortaleza 60135-218, Brazil; diego.martins@facpp.edu.br (D.M.d.P.); moisesparente98@gmail.com (A.M.P.d.P.); karenem@facpp.edu.br (K.E.C.); madiana.moreira@facpp.edu.br (M.M.M.); 2 Department of Chemistry, Federal University of Ceará, Fortaleza 60020-181, Brazil; diegolomonaco@ufc.br; 3 Department of Therapeutic Dentistry, I. M. Sechenov First Moscow State Medical University, 119146 Moscow, Russia; giovarruscio@me.com (M.G.); salvatore.sauro@uchceu.es (S.S.); 4 Dental Biomaterials and Minimally Invasive Dentistry, Department of Dentistry, Cardenal Herrera-CEU University, CEU Universities, C/Santiago Ramón y Cajal, s/n., Alfara del Patriarca, 46115 Valencia, Spain

**Keywords:** dentin, lignin, cardanol, proanthocyanidin, collagen cross-linking, dentistry

## Abstract

To evaluate the biomodification ability of lignin used as pre-treatment in human dentin before the application of an etch-and-rinse adhesive. Experimental hydroethanolic solutions with different cross-linking agents were used: 6.5% proanthocyanidins (PAC, from grape-seed extract); 2% cardanol (CARD, from cashew-nut shell liquid); lignin (LIG, from eucalyptus) at 1, 2 or 4% concentrations. The negative control (NC) was ethanol 50 v%. Extracted molars were prepared, and dentin microtensile bond strength (μTBS) was evaluated after 24 h water storage or 10,000 thermocycling aging. Further specimens were processed for SEM nanoleakage, micropermeability confocal microscopy evaluation and in situ degree of conversion (DC) through micro-Raman spectroscopy. Demineralized dentin sticks were submitted to a three-point bending test to evaluate the elastic modulus (E) before and after 1 min biomodification using the tested solutions. Moreover, it was also evaluated the mass changes and hydroxyproline (HYP) release after 4-weeks of water storage. Vibrational collagen crosslinking identification was evaluated through micro-Raman spectroscopy. The results were analyzed by analysis of variance (ANOVA) and Tukey’s test (α = 0.05). A significant reduction in μTBS was observed in groups NC (*p* < 0.001) and CARD (*p* = 0.026). LIG-4% showed no significant reduction in μTBS after aging (*p* = 0.022). Nanoleakage micrographs showed hybrid layer protection with all agents, but reduced micropermeability was attained only with lignin. Polymerization was negatively affected in the presence of all tested cross-linking agents, except LIG-1%. Lignin and cardanol increased the dentin E values, but only lignin reduced the mass loss in dentin specimens. Effective collagen crosslinking (1117 cm^−1^ and 1235 cm^−1^) was detected for all agents. HYP release was significantly lower with LIG-1% than NC (*p* < 0.001). Lignin was able to perform collagen cross-linking and prevent the degradation of unprotected dentin collagen, thereby improving the bonding performance of the composite restorations performed in this study.

## 1. Introduction

In order to attain a durable bonding in direct and indirect restorations, the establishment of a stable and well-sealed hybrid layer is essential [1]. Such a hybrid structure relies on an entangled mesh of resin monomers and collagen fibrils, which promotes micromechanical interlocking [1]. Nevertheless, due to the hydrated nature of demineralized [2], it results quite difficult to obtain a proper infiltration of hydrophobic monomers; several collagen fibrils are left unprotected, triggering bonding failures and the reduction of bonding longevity [3,4].

In modern restorative dentistry, many strategies have been advocated for dentin biomodification via collagen cross-linking [5] by means of enzymatic and non-enzymatic chemical approaches [6]. All these approaches have been demonstrated to improve the tensile strength and elastic modulus of demineralized dentin [4], as well the durability of composite restorations, since they reduce the proteolytic degradation within the hybrid layer induced by activated endogenous matrix metalloproteinases (MMPs) [3,7,8,9,10]. The biomodification strategy relies on the application of synthetic or natural compounds in dentin during restorative procedures [9]. Chemical agents, such as glutaraldehyde and carbodiimide can achieve a high degree of cross-linking in collagen, but their clinical application remains questionable due to potential cytotoxic issues [6,9,11].

Natural cross-linkers may sound more attractive as they originate from renewable and sustainable sources, with further interest in a global outlook [12,13]. Most of these agents are polyphenols, especially proanthocyanidins (PACs) from grape-seed extract (Vitis vinifera); this is highlighted for noteworthy outcomes as demonstrated in several investigations [6,10,14,15]. Particular mechanisms for collagen cross-linking are based on the formation of hydrogen, covalent bonds as well as hydrophobic interactions [6,12,16]. More recently, the long-carbon chain in cardanol (CARD) and cardol, obtained in industrial residue of cashew nut (Anacardium occidentale) production, demonstrated promising biomodification capacity [13,17].

Based on sustainability evidence, lignin eucalyptus (LIG), a polyphenol-rich natural polymer, represents the main component of secondary plant cells’ walls, which supplies rigidity to vegetal architecture by cross-linking cellulose and hemicellulose [18,19]. It can be obtained from black liquor produced during wood cooking in the industrial production of paper. Despite its promising chemical structure and high production worldwide, as per our knowledge, lignin was never investigated in adhesive dentistry [20].

Thus, the aim of this study was to evaluate the biomodification ability of lignin used as pre-treatment in human dentin before the application of an etch-and-rinse adhesive. The hypothesis of this study was that lignin would attain a similar cross-linking potential to PAC and CARD due to its favorable chemical structure characterized by a large number of hydroxyls able to bind to dentin collagen.

## 2. Materials and Methods

### 2.1. Preparation of Biomodification Solutions

**Figure 1:**
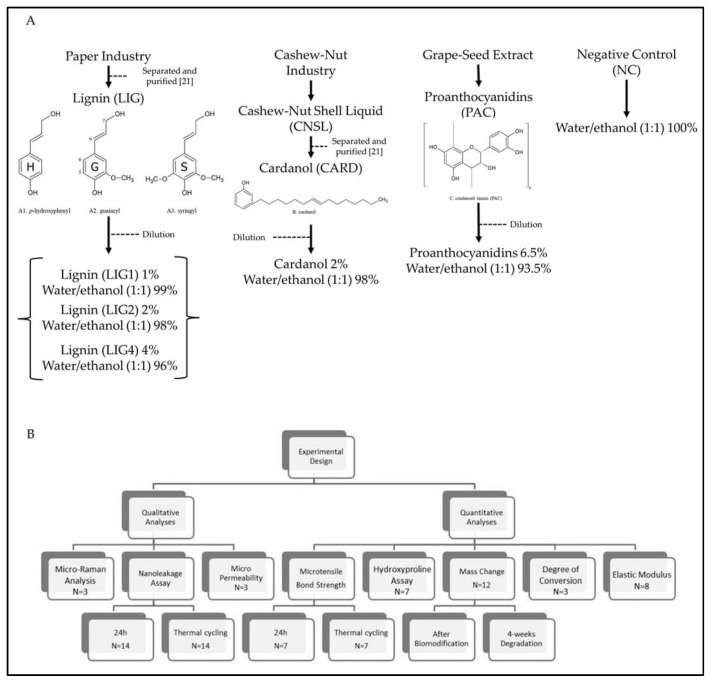
(**A**) Chemical structures of biomodification agents surveyed and steps diagram to the preparation of biomodification solutions [21]. A. Mono-lignol precursors of structural units forming the polymeric lignin molecule; B. Chemical structure of purified cardanol; C. Major structure of proanthocyanidins (PAC) from grape seed extract. (**B**) Diagram of experimental design with all experiments and number of specimens.

Cardanol (CARD) was obtained from cashew nut shell liquid, donated by Amêndoas do Brasil LTDA (Fortaleza, Brazil). CARD and LIG were extracted and purified by the methods described in the supplement appendix [21]. Cardanol was diluted in water/ethanol (1:1) at 2 wt% concentration. PAC solution was prepared by dissolving 6.5 wt% grape-seed extracts (Meganatural Gold, Madera, CA, USA) in water/ethanol (1:1) with 5 min stirring at 25 °C and double-filtering. LIG was supplied by the paper industry Suzano SA (Limeira, Brazil). It was diluted in water/ethanol (1:1) at 1 wt%, 2 wt% and 4 wt% concentrations. All solutions were buffered to pH 7.2. The hydroethanolic solution was employed to standardize the dissolution of CARD and LIG, which possess low solubility in distilled water. As a negative control (NC), the water/ethanol (1:1) solution without a biomodification agent was used. CARD and LIG were characterized, according to Moreira et al. (2017) [13], by Nuclear Magnetic Resonance (NMR). The chemical structures of the cross-linking agents employed in this study are shown in Figure 1.

### 2.2. Bonding Procedures

A total of 140 extracted human third molars were used in this study under the approval of the institutional Ethics Committee (protocol 011133/2018). Medium dentin surfaces were obtained by sectioning occlusal enamel with a diamond saw in a cutting machine (Cutmaster, Londrina, Brazil). Exposed dentin surfaces were abraded with wet 320-grit SiC papers for 30 s to standardize the smear layer.

The dentin was etched with 37% phosphoric-acid gel (Condac37, FGM, Joinville, Brazil) for 15 s and rinsed with distilled water for 30 s. The biomodification solutions were immediately applied for 60 s and rinsed for 20 s with distilled water. Dentin was left moist, and the two-step etch-and-rinse adhesive Optibond S (Kerr, Orange, CA, USA) was actively applied for 20 s, gently air-dried for 3 s and light-cured for 20 s with LED unit Valo (1200 mW/cm^2^, Ultradent, South Jordan, UT, USA). Two 2 mm-thick layers of resin composite (Opallis, FGM, Joinville, Brazil) were placed and individually light-cured for 40 s. Bonded teeth were stored in distilled water for 24 h at 37 °C.

### 2.3. Microtensile Bond Strength (µTBS) and Failure Pattern

Bonded teeth (*n* = 7) were longitudinally sectioned in resin-dentin sticks with a 1 mm² cross-sectional area. Half of the sticks per tooth were tested immediately, and the other half was subjected to thermo-cycling aging (1000 cycles; 30 s in 5 °C and 30 s in 55 °C with 10 s interval) (Huber-Mechatronik TC45SD, SD Mechatronik, Feldkirchen-Westerham, Germany) [22,23]. The test was performed by attaching the resin-dentin sticks in Geraldeli’s jigs with cyanoacrylate cement, adapted in a microtensile device (OM-100, Odeme, Luzerna, Brazil) and tested until failure with 0.5 mm/min crosshead speed. Prior to the test, each stick had a cross-sectional area measured with a digital caliper to obtain the bond strength in megapascals (MPa) [24]. Pre-test fractures were not often found and were included as 0 MPa.

All fractured sticks were examined by steromicroscopy (60× magnification, Stereo-zoom S8, Leica, Heidelberg, Germany) to identify failure patterns, classified as adhesive, cohesive in dentin, cohesive in composite or mixed.

### 2.4. Nanoleakage Survey

Two resin-dentin sticks from each subgroup were assessed for silver nanoleakage according to the protocol of Tay et al. (2002) [25], using a 50% ammoniacal silver nitrate solution. Briefly, specimens were immersed in tracer silver solution for 24 h in darkness, rinsed with distilled water and immersed in photodeveloping solution for 8 h under fluorescent light. They were then embedded in epoxy resin and polished with SiC papers up to 4000-grit and 1-µm diamond paste (Buehler, Lake Bluff, IL, USA) in polishing cloths. The specimens were cleaned for 5 min in an ultrasonic bath after each polishing step and dehydrated for 24 h in a silica gel incubator at 37 °C. They were gold-sputter coated and analyzed using field-emission-gun scanning electron microscopy (Quanta FEG, FEI, Amsterdam, The Netherlands) in backscattered electron mode.

### 2.5. Micropermeability Assay

Eighteen third molars (*n* = 3) were restored as aforementioned, using adhesive doped with 0.1 wt% Rhodamine B (Sigma-Aldrich, St. Louis, MO, USA). Bonded teeth were perfused with 0.3 wt% aqueous fluorescein (Sigma-Aldrich) solution under 15 cm H_2_O simulated pulpal pressure for 3 h. Thereafter, they were cut into 1 mm-thick slices, polished with 2000-grit wet SiC papers and ultrasonicated for 2 min. Specimens were observed in confocal-laser scanning microscopy (LSM 710, Leica) following the setup proposed by Feitosa et al. (2014) [26].

### 2.6. In Situ Degree of Conversion (DC)

To investigate the influence of each biomodification agent on adhesive resin polymerization, the protocol proposed by Navarra et al. (2016) [27] was followed. In summary, restorative procedures were performed as previously described, and bonded teeth were then cut into 1 mm-thick resin-dentin slabs. The specimens were positioned in a micro-Raman spectrophotometer (Xplora, Horiba, Paris, France) with a 100× magnification lens (Olympus, London, UK) to obtain a 1µm-beam diameter which was positioned in a hybrid layer. The ratio of vibrational intensities of aliphatic C=C from methacrylate (1639 cm^−1^) and the internal standard aromatic C=C (1609 cm^−1^) were obtained from uncured adhesive and cured adhesive within hybrid layers. DC was calculated following the formula: DC = (1–R-cured/R-uncured) × 100, where R is the ratio of peak heights of 1639 cm^−1^ and 1609 cm^−1^ vibrations. The analysis was performed in three slabs for each bonded tooth, and the results were averaged to obtain one statistical unit. Three bonded teeth per group were tested (*n* = 3).

### 2.7. Elastic Modulus (E)

A further 48 extracted molars were cut to obtain middle dentin sticks (1 mm-thick, 1 mm-width and 7 mm-length) [13]. The specimens were demineralized in 10% phosphoric acid for 5 h and then randomly distributed based on the five biomodification solutions and the negative control used in this study(*n* = 12). A three-point bending test was performed in untreated specimens (baseline) and those after 60 s immersion in each solution using a universal testing machine (Instron 3345, Instron Inc., Canton, OH, USA) with 5 N load cell, 0.5 mm/min crosshead speed. Elastic modulus was calculated by the software (Bluehill version 3.72 LE, Instron) after 1 mm displacement [10].

### 2.8. Mass Change (Wmc) and Biodegradation Rate (R)

The same demineralized dentin specimens used for the elastic modulus evaluation (*n* = 12) were also weighed using an analytical scaler (0.01 mg precision, AUX-220, Shimadzu, Tokyo, Japan) before (M1) and after (M2) the immersion in solutions. Prior to measurement, they were individually dehydrated for 24 h in a vacuum desiccator with silica gel at 25 °C. Mass change (Wmc%) was determined by the percentage of gain/loss of mass of each specimen [12].

The treated dentin specimens (*n* = 12) were then individually stored in 1.5 mL deionized water for 4 weeks to promote biodegradation after elastic modulus and mass change surveys. After such a period, specimens were dehydrated as aforementioned and weighed (M3). The protocol [12,13] was used to assess the percentage of biodegradation rate by mass loss.

### 2.9. Hydroxyproline Assay (HYP)

The storage solutions of treated dentin specimens after the 4-week storage period were collected to obtain three solutions of 6 mL each. Aliquots of supernatant were collected for assessment with an HYP assay kit (Sigma-Aldrich) following the manufacturer’s instructions. The final HYP-traced solutions were evaluated by UV-Vis spectroscopy (Ultrospec 1100 Pro, Amershan Biosciences, Little Chalfont, UK) with 550 nm wavelength [16] to obtain absorbance, which was transformed to HYP concentration by means of standard curve solutions supplied in the kit.

### 2.10. Micro-Raman Cross-Linking Identification

Further demineralized dentin sticks (*n* = 3) were investigated before and after 1-minute immersion in experimental solutions with the micro-Raman spectrophotometer (Xplora, Horiba) with 3.2 mW laser power and 632 nm wavelength using 10 s acquisition time and 3 accumulations. The spectra range was 700–1800 cm^−1^ to survey peaks and shoulders at 1117 cm^−1^ and 1235 cm^−1^ assigned to dentin collagen cross-linking [13].

### 2.11. Statistical Analysis

A Shapiro–Wilk normality test was used to assess normal distribution. After passing this test (*p* > 0.05), data were statistically analyzed by ANOVA or repeated-measures ANOVA and Tukey’s post hoc test (α = 5%).

## 3. Results

The outcomes obtained during the µTBS test, along with further quantitative experiments, are depicted in Table 1. NC and LIG1 achieved the highest initial results without a significant difference between them (*p* = 0.059). All further groups showed statistical differences from each other. After thermocycling, NC (*p* < 0.001) and CARD (*p* = 0.026) dropped the bond strength significantly, whilst LIG4 increased (*p* = 0.022). Further agents (LIG1, LIG2 and PAC) maintained stable µTBS (*p* > 0.05) after aging. Most fractures were adhesive for all groups.

**Figure 2:**
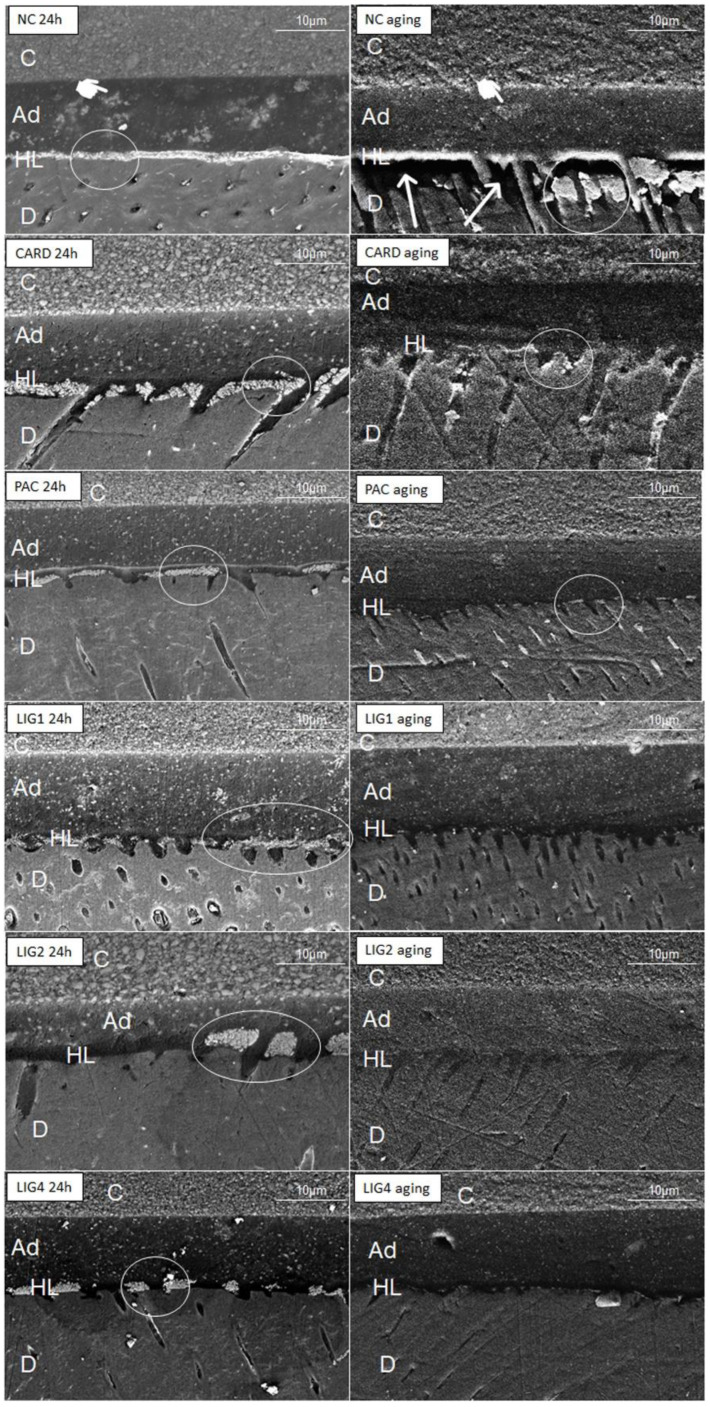
SEM images of the bonded interface of resin-dentin specimens assayed 24 h and after thermocycling aging. The pointers depict water-trees into the adhesive layer, arrows represent a fracture, and circles highlight silver infiltration into the hybrid layer. NC: Negative Control; CARD: Cardanol; PAC: Proanthocyanidin; LIG: Lignin; C: composite layer; Ad: adhesive layer; HL: hybrid layer; D, dentin. NC showed water-trees and striking silver uptake both in both 24 h and after aging. CARD and PAC showed areas of silver uptake both in 24 h and after aging. Water-trees were found only with PAC after aging. LIG1, LIG2 and LIG4 showed similar areas of silver uptake in HL but very little nanoleakage after aging.

**Figure 3:**
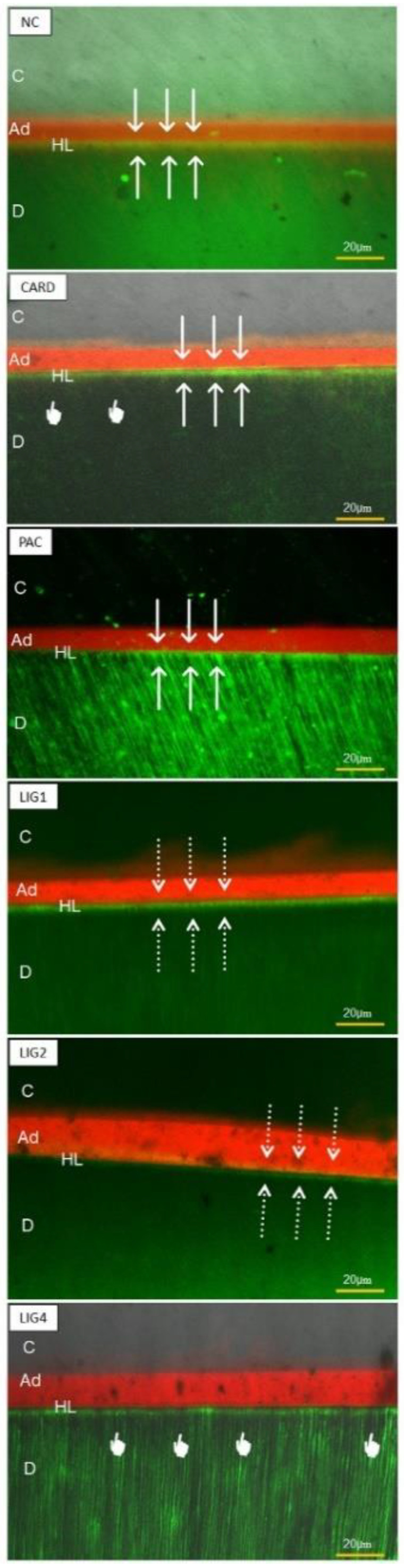
Confocal microscopy images representing the most common micropermeability outcomes at the bonding interfaces of resin-dentin specimens tested in this study. The pointers represent the protected hybrid layer; the arrows represent large infiltration of fluorescein; dotted arrows represent little infiltration of hybrid layer. In general, most prominent micropermeability was observed in negative control and PAC-treated specimens, whilst less micropermeability was found in those specimens treated with 4% lignin. NC: Negative Control; CARD: Cardanol; PAC: Proanthocyanidin; LIG: Lignin; C: composite layer; Ad: adhesive layer; HL: hybrid layer; D: dentin.

Nanoleakage micrographs are shown in Figure 2. The negative control presented more intense silver uptake at the adhesive interface both at 24 h and after aging. Lignin in all concentrations provided similar nanoleakage only in a hybrid layer, with very few silver deposits after aging. CARD and PAC attained intermediary nanoleakage. Figure 3 show some representative images of micropermeability. The highest micropermeability was observed in NC and PAC-treated specimens, whereas a less permeable interface was attained with LIG-4%.

The presence of most of the biomodification agents tested in this study decreased the DC (Table 1), except for LIG1, which attained a significantly lower DC than the negative control (*p* < 0.05). No statistical difference (*p* = 0.695) was found between NC and LIG1. Regarding elastic modulus (E), LIG-4% yielded the highest percentage increase on flexural modulus in comparison to the other tested groups (Table 1). Conversely, the absence of biomodification agents in NC promoted E reduction.

Lignin in lower concentrations (LIG1 and LIG2) achieved the best mass gain (Table 1) after 4-week water storage, with a significant difference between the negative control and PAC. HYP quantifications are shown in Table 1. Statistical lower HYP release was attained with LIG1 (0.33 ± 0.01 µg/mL) in comparison to NC (0.96 ± 0.12 µg/mL, *p* < 0.001). The other tested agents showed intermediate HYP-release outcomes.

**Figure 4:**
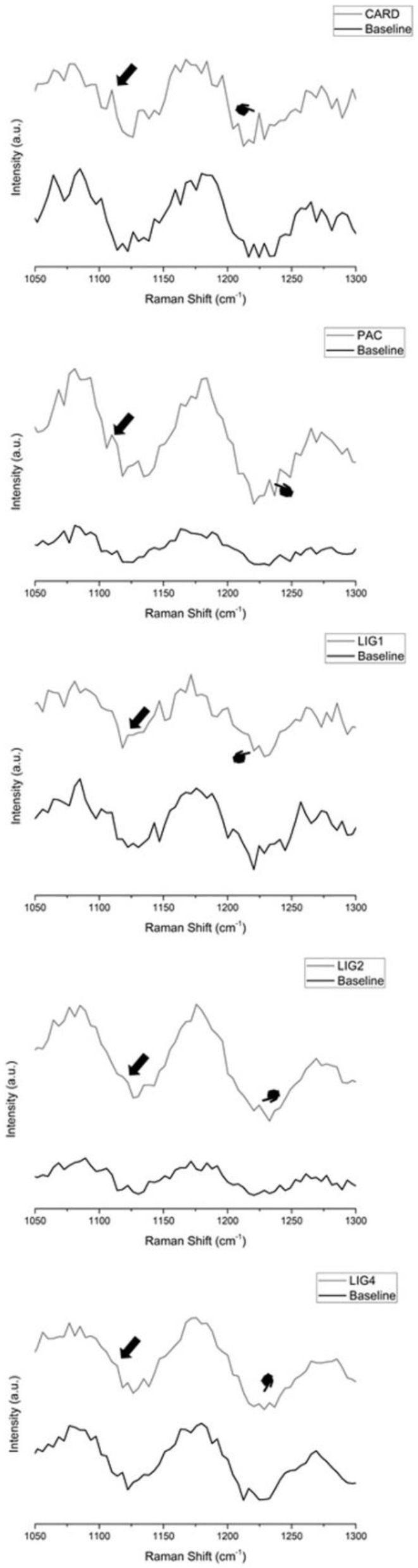
Vibrational Micro-Raman spectra of same specimens before (Baseline) and after 1 minute biomodification treatment. All agents induced emergence of shoulder at ~1117 cm^−1^ (black arrow) and increase of Amide III peak at 1235 cm^−1^ (pointers) which demonstrates collagen crosslinking. CARD: Cardadol; PAC: Proanthocyanidin; LIG: Lignin.

Raman spectra of the cross-linking formation are reported in Figure 4. All biomodification agents were characterized by the appearance of a shoulder at approximately 1117 cm^−1^ and an increase of the peak at 1235 cm^−1^, which confirm collagen cross-linking.

## 4. Discussion

The antiviral, antineoplastic, anti-inflammatory and osteogenic activities of lignin were previously demonstrated [18,20]. The present investigation evaluated for the first time the ability of lignin (from Eucalyptus) used as a biomodification agent to improve the biomechanical and biochemical properties of demineralized human dentin, as well as the bonding stability of hybrid layers created with a simplified etch and rinse dentin bonding agent. Two previously proven biomodification agents (PAC from Vitis vinifera and cardanol from Anacardium occidentale) were included in the study design for comparison. The hypothesis tested in this study needs to be rejected because only lignin used in lower concentrations attained better overall outcomes than PAC and CARD.

Lignin is an important biopolymer discarded by biofuel and cellulose companies [19]. Furthermore, the lignin subproducts in bio-refineries are also used to obtain aromatic biopolymers with antioxidant and antimicrobial capacity. Therefore, lignin represents a promising raw material for application in several medical fields [28]. All plants possess a high amount of lignin (~25% of biomass), and it is the second most abundant polymer worldwide [29]. In contrast to cellulose, lignin rarely presents a monomeric structure [30]. However, its chemical structure is not fully known due to alterations undergone during wood processing. Lignin’s mainframe is phenyl-propane, linked to a benzene ring with a variable number of hydroxyl (10%) and methoxyl groups (90%). All these functionalities comprise distinct phenols in an aromatic polymer complex of monomeric sub-unities, such as p-hydroxyphenyl (H), guaiacyl (G) and syringyl (S) [19,30]. Furthermore, structural complexity increases depending on internal cross-links, which differ in each plant species [29].

A current trend in restorative dentistry advocates the biomimetic remineralization of dental hard tissues [9,31] and the biomodification of dentin with natural collagen cross-linkers [9]. The latter tends to increase the crosslinks within the mature collagen matrix by non-enzymatic reactions in order to strengthen the biomechanical properties of the dentine and drastically reduce its degradation [6], using plant-derived polyphenols with low toxicity [9]. PACs are considered condensed tannins with high protein affinity and antioxidant capacity; these are well investigated in functionalized biomaterials [32]. Cross-linking does not only depend on the number of hydroxyls but also on the entire molecular structure of PACs, including aromatic rings that trigger hydrophobic bonds [6,9,32].

A further biomodification agent employed herein was cardanol, from cashew-nut shell liquid, obtained as a subproduct of the cashew industry [33,34]. Cardanol is a long carbon-chain (15 carbons) phenol with high hydrophobicity and several sites for organic synthesis [17,34]. In dentistry, it was advocated as a suitable agent for the treatment of dentin hypersensitivity [17], which retains the ability to increase the elastic modulus of dentin and to reduce the biodegradation of demineralized dentin without color alteration [13].

The presence of all biomodification agents used in this study as therapeutic dentin primers after phosphoric-acid etching and prior to the application of a two-step etch-and-rinse adhesive reduced the initial bond strength (Table 1), except when LIG1 was used; such results are in accordance with those of Hass et al. (2016) [11]. This issue might occur due to a lack of monomer polymerization within the hybrid layer, as confirmed by DC results (Table 1). PACs are oligomeric molecules, constituted majorly by catechin, epicatechin and epigallocatechin sub-unities. Indeed, the large molecular size of PAC may impair optimal penetration in collagen mesh, resulting in lower µTBS. Conversely, lignin possesses a hetero-polymeric aromatic complex with three main units (Figure 1) characterized by hydroxyl and methoxyl functionalities [35], which likely bind to collagen providing adequate µTBS despite its large molecular size.

The aging protocol used in this study for the resin-dentin sticks was 10,000 thermo-cycling aging [22,23]. Conversely, an aging protocol based on simulated pulpal pressure (SPP) could induce an excessive release of biomodification from the dentin interface, depending on molecular weight [19]. However, Perote et al. (2015) [36] demonstrated that both protocols (SPP and thermocycling) may produce similar bond strength reduction. Most experimental groups maintained the µTBS values, thereby proving reliable bond stability as a consequence of dentin biomodification. This was also confirmed by the presence of Raman peaks at 1117 cm^−1^ and 1235 cm^−1^ (Figure 4) [6,13]. Indeed, improved dentin sealing, as observed in confocal microscopy, was mainly achieved by PAC, CARD and LIG4 (Figure 3).

SEM micrographs obtained after aging the specimens (Figure 2) depicted little water sorption and few voids within hybrid layers created when employing the tested biomodification agents, especially with lignin. The fact that CARD decreased the µTBS values after aging might be explained by its molecular structure, which comprises solely one hydroxyl able to create hydrogen bonds. We speculate that such situation facilitated the leaching of biomodification agent from the dentin over time. However, CARD promoted a noteworthy increase in the elastic modulus of the dentin; this may be due to its linear and low-molecular-weight chemical structure (Figure 1). Furthermore, the long and hydrophobic carbon chain may guarantee collagen protection during 4-week water storage [13,17].

Regarding LIG-4%, the excess of lignin may have provided an additional cross-linking effect over time, improving the µTBS to dentin. It was previously reported that the cross-linking of a biomodification agent is dose-dependent [37], corroborating the explanation about bond strength increase with LIG4. In the negative control, the absence of a biomodification agent demonstrated rapid interfacial degradation, mostly due to MMPs and resin-sparse unprotected collagen fibrils [38], resulting in approximately 40% bond strength loss after thermocycling.

Proanthocyanidins from grape-seed extract (Vitis vinifera) are classified as condensed tannin with B-type interflavonoid links, showing effectiveness in increasing the rigidity of demineralized dentin matrix higher than further non-galloylated compounds [38,39]. Nevertheless, this type of PAC is less stable in comparison with A-type ones [40] because galloyl-bonds are prone to undergo hydrolysis at 3-O-gallate ester during aging, which might be exacerbated by oxidation reactions [40]. Indeed, this may explain the loss of activity of PAC in augmenting elastic modulus and providing mass gain, yielding intermediary release of HYP (Table 1).

Higher concentrations of lignin clearly improved the elastic modulus of dentin collagen (Table 1), probably in a similar way as it occurs in plant cell walls, where the bonding between cellulose fibers and lignin is accomplished via covalent bonds [19]. Nonetheless, lignin at 1% achieved better outcomes of mass change during water storage and the least HYP release, thereby proving the efficacy in protecting demineralized dentin from degradation. Due to its branched and amorphous chemical structure, lignin in lower concentrations might better infiltrate the three-dimensional spaces within collagen fibrils. Moreover, thanks to several aromatic rings and to intra-molecular cross-links, lignin may provide stable collagen cross-linking for protection against hydrolysis, thermocycling and MMPs-mediated degradation [41].

## 5. Conclusions

Lignin can cross-link and reinforce demineralized dentin collagen, improving the bond stability of composite restorations and preventing collagen degradation, particularly when applied at low (1 wt%) concentrations; such a lower concentration will not affect the degree of conversion of dental adhesive resins. Future investigations should focus on further uses of lignin in dentistry.

## Figures and Tables

**Table 1:** Means (standard deviations) of quantitative results of various experiments.

Groups	µTBS (MPa) [Fracture Mode A/CD/CC/M]		DC	Modulus of Elasticity			Mass Change (%)		HYP (µg/mL)
	Immediate	Aging	(%)	Baseline (MPa)	Treated (MPa)	Variation (%)	After Biomodif	4-Weeks Degradation	Not-Demineralized 0.02 (0.01) ^D^
NC	51.2 (4.9) ^Aa^ [90/2/3/5]	36.8 (7.2) ^ABb^ [97/0/0/3]	75.7 (4.5) ^A^	10.6 (1.9) ^a^	4.1 (7.2) ^b^	−60.4 (23.2) ^C^	13.5 (6.7) ^C^	−34.4 (29.8) ^C^	0.96 (0.12) ^A^
LIG1	39.6 (3.3) ^ABa^ [98/0/0/2]	35.7 (7.4) ^Ba^ [92/0/0/8]	71.1 (4.3) ^AB^	3.6 (1.1) ^a^	4.3 (1.9) ^a^	22.8 (35.7) ^BC^	30.7 (8.6) ^A^	45.2 (13.2) ^A^	0.33 (0.01) ^C^
LIG2	38.1 (6.2) ^BCa^ [98/0/0/2]	37.7 (2.6) ^ABa^ [95/0/0/5]	64.1 (0.2) ^BC^	2.7 (0.8) ^b^	5.6 (2.2) ^a^	116.5 (74.5) ^A^	25.7 (8.7) ^AB^	42.9 (19.1) ^A^	0.53 (0.01) ^B^
LIG4	37.5 (3.0) ^BCb^ [99/0/0/1]	46.4 (5.6) ^Aa^ [92/0/3/5]	64.1 (6.1) ^BC^	3.6 (1.2) ^b^	8.4 (3.7) ^a^	177.6 (263.8) ^AB^	16.1 (11.5) ^BC^	27.3 (16.1) ^AB^	0.55 (0.09) ^B^
CARD	37.9 (4.0) ^BCa^ [100/0/0/0]	31.2 (5.5) ^Bb^ [97/2/0/1]	57.7 (2.3) ^C^	4.4 (1.0) ^b^	7.9 (3.9) ^a^	85.5 (96.7) ^AB^	21.4 (9,0) ^ABC^	20.3 (17.5) ^AB^	0.50 (0.07) ^BC^
PAC	30.3 (4.9) ^Ca^ [100/0/0/0]	30.2 (4.0) ^Ba^ [100/0/0/0]	56.8 (3.3) ^C^	7.4 (2.0) ^a^	8.6 (2.2) ^a^	17.1 (12.7) ^BC^	19.2 (5.4) ^ABC^	4.5 (33.0) ^B^	0.61 (0.06) ^B^

Lowercase letters represent statistical differences in rows and uppercase letters in columns (*p* < 0.05). NC: negative control; LIG: lignin; CARD: cardanol; PAC: proanthocyanidin. Fractures percentages-[adhesive/cohesive in dentin/cohesive in composite/mixed].

## Data Availability

The data will be available upon request by email.
